# Cyclodextrins for structural and functional studies of mechanosensitive channels

**DOI:** 10.1016/j.yjsbx.2021.100053

**Published:** 2021-11-07

**Authors:** Yixiao Zhang, Gabriella Angiulli, Boris Martinac, Charles D. Cox, Thomas Walz

**Affiliations:** a Laboratory of Molecular Electron Microscopy, The Rockefeller University, New York, NY, USA; b St Vincent's Clinical School, University of New South Wales, Sydney, New South Wales, Australia; c Molecular Cardiology and Biophysics Division, Victor Chang Cardiac Research Institute, Sydney, New South Wales, Australia

**Keywords:** Mechanosensitive channels, Single-particle cryo-electron microscopy, Electrophysiology, Beta-cyclodextrin

## Abstract

Mechanosensitive (MS) channels that are activated by the ‘force-from-lipids’ (FFL) principle rest in the membrane in a closed state but open a transmembrane pore in response to changes in the transmembrane pressure profile. The molecular implementations of the FFL principle vary widely between different MS channel families. The function of MS channels is often studied by patch-clamp electrophysiology, in which mechanical force or amphipathic molecules are used to activate the channels. Structural studies of MS channels in states other than the closed resting state typically relied on the use of mutant channels. Cyclodextrins (CDs) were recently introduced as a relatively easy and convenient approach to generate membrane tension. The principle is that CDs chelate hydrophobic molecules and can remove lipids from membranes, thus forcing the remaining lipids to cover more surface area and creating tension for membrane proteins residing in the membranes. CDs can be used to study the structure of MS channels in a membrane under tension by using single-particle cryo-electron microscopy to image the channels in nanodiscs after incubation with CDs as well as to characterize the function of MS channels by using patch-clamp electrophysiology to record the effect of CDs on channels inserted into membrane patches excised from proteoliposomes. Importantly, because incubation of membrane patches with CDs results in the activation of MscL, an MS channel that opens only shortly before membrane rupture, CD-mediated lipid removal appears to generate sufficient force to open most if not all types of MS channels that follow the FFL principle.

## Highlights

• Cyclodextrins (CDs) remove lipids from membranes and thus generate membrane tension.
• CDs can be used to activate ‘force-from-lipids’-gated mechanosensitive (MS) channels.
• CDs can activate MS channels for structural studies by single-particle cryo-EM.
• CDs can activate MS channels for functional studies by patch-clamp electrophysiology.

## Graphical abstract

**Figure:**
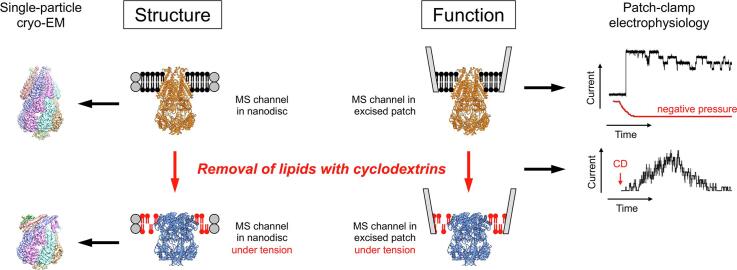

Membrane channels form transmembrane pathways that allow cells to exchange solutes with their environment. Unlike transporters, channels are passive conduits that enable solutes to cross the membrane down their electrochemical gradients. While some channels are constitutively open, most channels open only under specific conditions. Ligand-gated channels open upon binding of a ligand (Fig. 1, top left) and voltage-gated channels open upon changes in the membrane potential (Fig. 1, top right). Mechanically gated channels respond to forces that act upon them either through tethers that connect them to cytoskeletal or extracellular components (‘force-from-filament’; FFF) (Fig. 1, bottom left) or through changes in the transmembrane pressure profile of the surrounding lipid bilayer (‘force-from-lipids’; FFL) (Fig. 1, bottom right) (reviewed in Cox et al. (2019)). In addition, many channels can be regulated by more than one of these mechanisms as well as by changes in temperature.

**Fig. 1:**
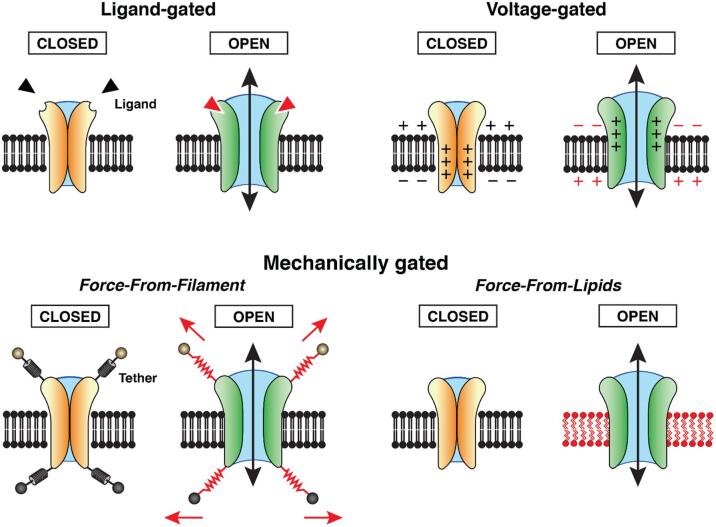
Different classes of gated channels. Gated channels are closed in the resting state and require a triggering event to open and allow solutes and/or ions to cross the membrane along their electrochemical gradients. Top left: Many channels open in response to the binding of a ligand. Top right: For voltage-gated channels, opening of the transmembrane channel depends on a change in the membrane potential that is sensed by charged residues in voltage-sensor domains. Bottom: Mechanosensitive (MS) channels react to mechanical force. This force can be transmitted to the MS channel through tethers that link the channel to components of the extracellular matrix or the cytoskeleton, known as the force-from-filament principle (FFF) (bottom left), or can be exerted on the MS channel by changes in the transmembrane pressure profile of the surrounding lipid bilayer, known as the force-from-lipid principle (FFL) (bottom right).

It has been mostly straightforward to elucidate the gating mechanism for ligand-gated channels, due to the simplicity of just having to add the ligand to understand the effect it has on the structure and function of the channel, but understanding the gating mechanism for FFL-based MS channels has proven more difficult. For functional characterizations, these channels are typically studied by patch-clamp electrophysiology, in which the channels are activated either by applying a negative pressure or by adding amphipathic molecules to the excised membrane patch (Martinac et al., 1990, Sukharev et al., 1993, Vásquez et al., 2008). In contrast, structures for MS channels were obtained mostly by X-ray crystallography of detergent-solubilized channels in the resting closed conformation, while structural information for channels in the open conformation remained sparse and relied mostly on mutant channels displaying an increased open probability (Wang et al., 2008) or on spectroscopy techniques, such as electron paramagnetic resonance spectroscopy (e.g., Perozo et al. (2002)) and Förster resonance energy transfer spectroscopy (e.g., Wang et al. (2014)). Structural information for MS channels in different functional states, such as sub-conducting, adapted or desensitized states, remained elusive. The problem for determining the structure of MS channels in different functional states, necessary to understand the intricacies of their molecular dynamics, is that the channels are gated by properties of the surrounding lipid bilayer, namely the transmembrane pressure profile. Until recently, the predominant approach to membrane protein structure determination was X-ray crystallography, which required the membrane protein to be extracted from the lipid bilayer, thus removing the lipid environment that underlies the regulation of FFL-based MS channels. A solution to this problem emerged with two important developments: (1) the introduction of membrane-scaffold protein (MSP)-based nanodiscs as an alternative way to stabilize membrane proteins (Civjan et al., 2003) and (2) the advent of direct electron detectors that transformed single-particle cryo-electron microscopy (cryo-EM) into the method of choice to determine membrane protein structures (De Zorzi et al., 2016, Cheng, 2018). These two developments laid the foundation for the introduction of cyclodextrins (CDs) as a novel and simple approach to activate FFL-based MS channels for structural and functional studies (Zhang et al., 2021, Cox et al., 2021).

CDs are macrocyclic oligosaccharides formed by five or more α-D-glucopyranoside units linked through α(1→4) glycosidic bonds (Fig. 2A). CDs adopt toroidal shapes with the primary and secondary hydroxyl groups extending from the smaller and larger openings, respectively (Fig. 2B). While the exterior of the toroid is hydrophilic, the interior is less hydrophilic than the aqueous environment and thus attracts hydrophobic guest molecules. The three naturally occurring CDs, α, β and γCD, are formed by six, seven and eight glucopyranoside subunits, respectively, and thus differ in the size of their hydrophobic cavities (Fig. 2C), resulting in their different affinities for guest molecules. CDs are widely used, including in the cosmetic, food, textile, separation science and pharmaceutical industries, and have been the topic of numerous recent reviews (e.g., Petitjean et al. (2021)). In biology, the different affinities of CDs have been exploited to either specifically remove cholesterol from plasma membranes of cells grown in cell culture using methylated βCD or to preferentially remove phospholipids using αCDs (Zidovetzki and Levitan, 2007 and references therein). Methylated βCD has also been used for detergent removal for two-dimensional crystallization of membrane proteins (Signorell et al., 2007).

**Fig. 2:**
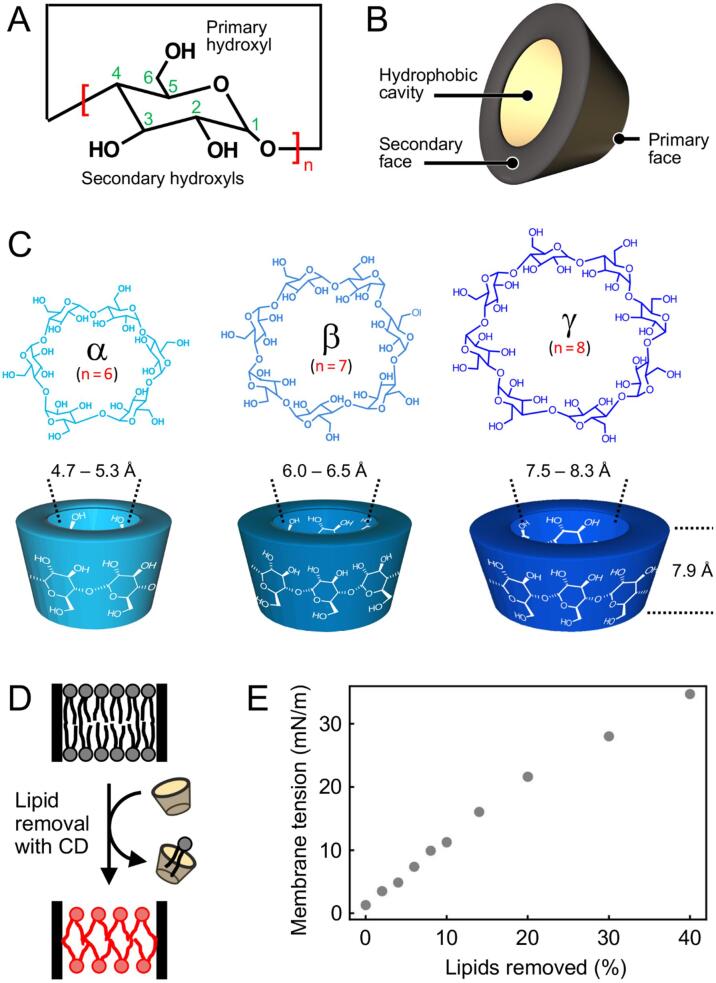
Cyclodextrins and their use to create membrane tension. (A) Chemical structure of the cyclodextrin subunit, an α-D-glucopyranoside, that forms α(1→4) glycosidic bonds with neighboring subunits. (B) CDs adopt toroidal shapes with an interior cavity that is less hydrophilic than the aqueous environment and thus attracts hydrophobic guest molecules. The primary hydroxyl groups of the glucopyranoside units extend from the smaller opening of the toroid and the secondary hydroxyl groups from the larger opening. (C) Chemical structure (top) and 3D representation (bottom) of the three naturally occurring CDs. α, β and γCD are formed by six, seven and eight glucopyranoside units, respectively, and feature increasingly larger hydrophobic cavities. (D) CDs remove lipids from membranes. If the surface area covered by the membrane does not change, each remaining lipid must cover more surface area, creating tension in the membrane. Note that CDs will only create membrane tension if the removed lipids are not being replaced from a lipid reservoir. (E) Graph showing the increase in membrane tension as lipids are gradually removed. The graph was obtained from molecular dynamics simulations of a bilayer containing 200 DOPC lipids, from which two lipids were removed from each leaflet at a time (for more details, see Zhang et al., 2021). Panels A to C adapted from (Crini et al., 2018); panel E is from (Zhang et al., 2021).

CDs can also be used to extract phospholipids from membranes. If the surface area occupied by the membrane does not change, each remaining lipid will have to cover more surface area, which will increase the tension in the membrane (Fig. 2D). Molecular dynamics simulations provided an estimate of the increase in membrane tension with removal of lipids from a bilayer (Zhang et al., 2021) (Fig. 2E). Since lipid extraction will result in a change in the transmembrane pressure profile, incubation of membranes with CDs should result in potent activation of FFL-based MS channels, a notion that was demonstrated with bacterial MS channels (Zhang et al., 2021, Cox et al., 2021).

*E. coli* expresses members of the Mechanosensitive channel of Small conductance (MscS) and the Mechanosensitive channel of Large conductance (MscL) families. X-ray crystal structures for the homoheptameric MscS have been determined in both the closed and open states and provided the first insights into the conformational changes underlying channel gating (e.g., Wang et al., 2008, Bass et al., 2002, and Lai et al. (2013)). However, the precise molecular mechanism of how membrane tension opens the channel remained unclear. More recently, the “lipids-move-first” model has been posited as a molecular interpretation of the FFL principle. Here, lipids that fill hydrophobic pockets between the subunits dissociate first with membrane tension, allowing channel opening presumably through the release of stored elastic strain energy (Bavi et al., 2017, Pliotas et al., 2015). The direct influence of lipids on MscS activity (Nomura et al., 2012) and their proposed intimate role in MscS gating underscored the need to obtain structures of MscS in the context of a native membrane environment, which was accomplished by single-particle cryo-EM studies of MscS reconstituted into nanodiscs. The resulting density maps revealed lipids associated with the channel and refined our understanding of how the channel sits in the membrane (Rasmussen et al., 2019, Reddy et al., 2019). The reconstitution of MscS into nanodiscs also opened the way to using CDs to remove lipids from the nanodiscs and thus to study the structure and dynamics of MscS in a membrane under tension (Zhang et al., 2021).

MscS was first reconstituted into a nanodisc with dioleoyl phosphatidylcholine (DOPC) lipids and the resulting density map showed the channel in the expected closed resting conformation as well as density for associated lipids seen in the previous two cryo-EM maps of MscS in nanodiscs (Rasmussen et al., 2019, Reddy et al., 2019). Based on their position and characteristics these lipids were termed the “pore” “pocket” and “gatekeeper” lipids (Zhang et al., 2021) (Fig. 3A). Incubation of the MscS-containing nanodiscs with βCD resulted in increasing aggregation of the sample but enough nanodiscs remained intact and could be purified by size-exclusion chromatography for structure determination by single-particle cryo-EM. The resulting map revealed the channel in a previously unknown conformation in which the pore is too narrow to allow ion conduction. This map also did not show much density representing the pocket lipids (Fig. 3B). This structure was most consistent with MscS in a desensitized state. Hence βCD-mediated lipid removal created tension in the membrane presumably opening the channel but because the tension in the membrane persisted after activation the channel transitioned further into the desensitized state.

**Fig. 3:**
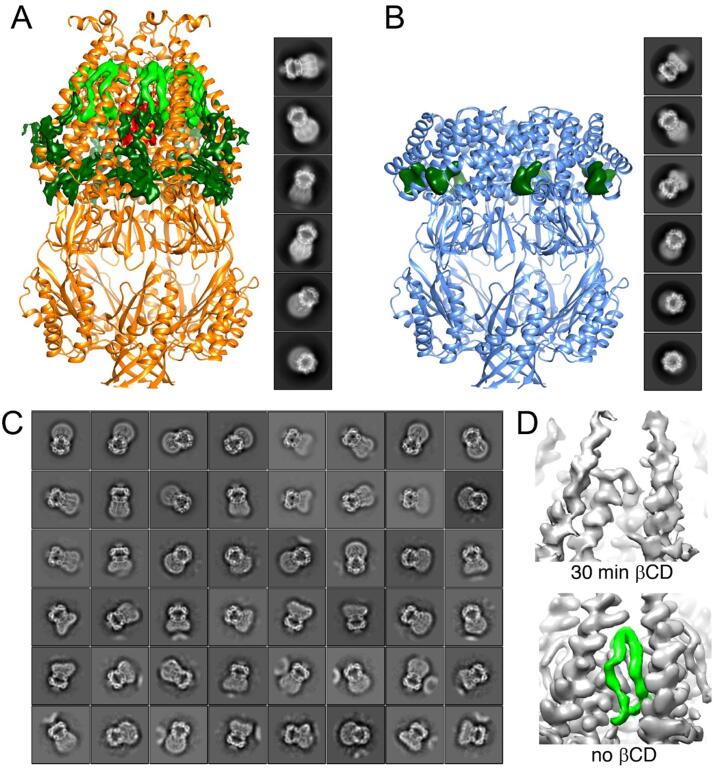
The use of βCD for the structural study of the bacterial MS channel MscS. (A) Cryo-EM structure of MscS reconstituted with DOPC into an MSP-stabilized nanodisc, showing the different types of lipids associated with the channel. The channel is shown in ribbon representation and density representing associated lipids is color coded: red, pore lipids; dark green, pocket lipids; light green, gatekeeper lipids. The inset to the right shows representative 2D-class averages. (B) After extensive incubation of MscS-containing nanodiscs with βCD, the resulting membrane tension causes the channel to transition into the desensitized state, in which the transmembrane domain is compressed, the pore is closed, and only little density is seen representing pocket lipids. The channel is shown in ribbon representation and remaining density for pocket lipids is shown in dark green. The inset to the right shows representative 2D-class averages. (C) All 2D-class averages resulting from cryo-EM analysis of nanodiscs containing the non-desensitizing Gly113Ala MscS mutant after incubation with βCD feature a smeared-out transmembrane domain (compared to averages in panels A and B), suggesting that, in the context of a lipid bilayer, MscS in the open state does not adopt a single, defined conformation. (D) After only a short incubation of MscS-containing nanodiscs with βCD, one of the cryo-EM density maps showed the channel still in the closed conformation but lacking density for the gatekeeper lipid, suggesting that membrane tension causes this lipid to dissociate before the channel transitions to the open conformation. The side length of individual averages in panels (A), (B) and (C) is 21.6 nm. (For interpretation of the references to color in this figure legend, the reader is referred to the web version of this article.)

To visualize MscS in an open state, the non-desensitizing Gly113Ala MscS mutant (Akitake et al., 2007) was reconstituted into DOPC nanodiscs and incubated with βCD. After size-exclusion chromatography, the nanodisc-embedded Gly113Ala MscS was visualized by cryo-EM, but image processing yielded only 2D averages with smeared-out transmembrane domains (Fig. 3C) and none of the 3D maps showed a well-defined transmembrane domain. Cryo-EM analysis of nanodiscs containing the Ala106Val MscS mutant, which has a very high open probability and was used to determine the X-ray structure of MscS in the open state (Wang et al., 2008), also failed to generate density maps with a well-defined transmembrane domain. The failure to resolve the open MscS conformation seen in the X-ray structure of the detergent-solubilized channel thus does not appear to be a failure of βCD to induce MscS to adopt the open state but instead suggests that, in the context of a lipid bilayer, MscS in the open state does not adopt a single, defined conformation. Furthermore, when nanodiscs containing wild-type MscS were incubated with βCD for a shorter time, most 2D averages and 3D maps showed MscS with a smeared-out transmembrane domain, but one map still showed MscS in the closed conformation. This map lacked density for the gatekeeper lipid (Fig. 3D), indicating that this lipid dissociates first, before the channel then transitions to the open conformation. This result not only supports the lipids-move-first mechanism of MscS gating but also strongly suggests that changing the incubation time (and/or the βCD concentration) can be used for time-resolved structural studies of mechanosensation by MS channels and other membrane- associated mechanoreceptors.

To confirm that the MscS conformation after incubation with βCD indeed represents the channel in the desensitized state, the effect of βCD was also functionally analyzed by using patch-clamp electrophysiology on MscS reconstituted into azolectin liposomes (Zhang et al., 2021, Cox et al., 2021). In the absence of negative pressure or βCD, no channel activity was observed (Fig. 4A; top trace). Perfusion of the excised patches with βCD elicited robust MscS activity and in patches that did not rupture the MscS current subsequently declined to baseline (Fig. 4A; middle trace), showing that prolonged incubations with βCD indeed result in channel closure. Application of negative pressure to these patches failed to elicit any further MscS activity (Fig. 4A; bottom trace), demonstrating that MscS adopted a desensitized rather than an adapted state.

**Fig. 4:**
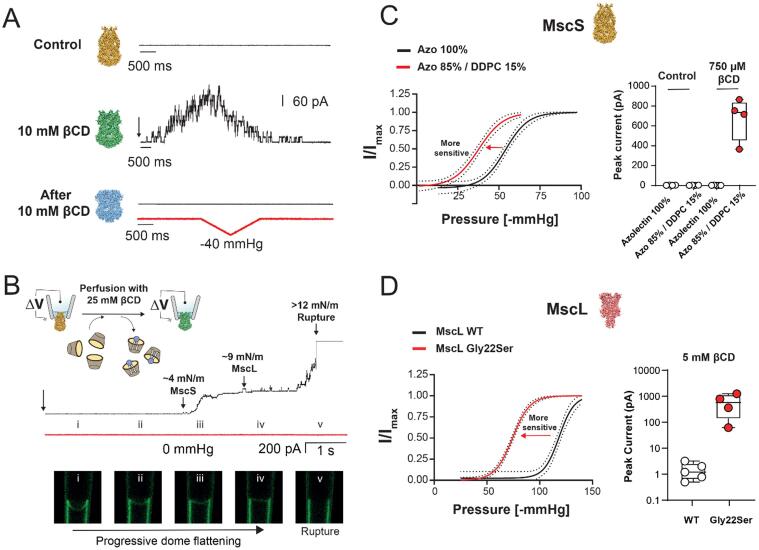
The use of CDs for the functional study of bacterial MS channels. (A) Electrophysiological recording of an excised azolectin membrane patch containing MscS. Top trace: Control that shows that no current is observed in the absence of negative pressure or βCD, demonstrating that MscS is closed in the resting state. Middle trace: Perfusion with 10 mM βCD elicits robust MscS activity that subsequently declines to baseline, demonstrating that βCD triggers MscS to open and then to close again. Bottom trace: Subsequent application of negative pressure no longer elicits any further MscS activity, demonstrating that MscS has adopted the desensitized state. (B) Electrophysiological recording of an excised azolectin membrane patch containing MscS and MscL after addition of 25 mM βCD. Top panel: The trace shows activation of first MscS (activation threshold ∼ 4 mN/m) and then MscL (activation threshold ∼ 9 mN/m). Bottom panels: MS channel activation coincides with progressive flattening of the patch dome due to increasing membrane tension, which finally results in the rupture of the azolectin membrane patch at a tension that exceeds ∼ 12 mN/m. (C) The left panel shows that less membrane tension is required to open MscS in liposomes that contain 15% DDPC than MscS in liposomes that contain only azolectin (Azo). In liposomes containing 15% DDPC, 750 µM βCD can instigate MscS opening while the same concentration does not induce MscS opening in liposomes containing only azolectin. The right panel shows summary data for the peak MscS currents elicited by 750 µM βCD in the two lipid environments. (D) The left panel shows that the Gly22Ser MscL mutant requires less membrane tension to open than wild-type (WT) MscL. In azolectin liposomes, Gly22Ser MscL is activated robustly in response to perfusion of 5 mM vCD, while the same concentration of βCD does not induce opening of WT MscL. The right panel shows summary data for the peak MscL currents for the WT channel and the Gly22Ser mutant elicited by 5 mM βCD. Panel A is adapted from (Zhang et al., 2021). Panels B-D are adapted from (Cox et al., 2021).

Additional work was performed to further characterize the use of CDs to study the function of MS channels, establishing that all tested CDs (α,βand γCD as well as the methylated derivative of βCD) can be used to activate MscS and that this activation depends on CD-mediated lipid removal (Cox et al., 2021).

To test whether CDs can also activate other MS channels, excised membrane patches containing both MscS and MscL were perfused with βCD (Fig. 4B). MscS were the first channels to open and as the MscS current saturated, MscL began to open, whose current amplitude is three times that of MscS. Over time more MscL opened until the tension caused by βCD-mediated lipid removal became too great and caused the membrane patch to rupture. Since MscL only opens at very high membrane tension, just before membranes rupture, CD-mediated activation of this channel suggests that CD-mediated lipid removal can be used to activate most if not all FFL-gated MS channels.

Finally, two sets of experiments showed that the activating CD concentration scales with the tension sensitivity of the MS channel. For MscS, tension sensitivity is affected by the membrane environment, and incorporation of 30% didecanoyl phosphatidylcholine (DDPC) to an azolectin membrane resulted in MscS showing spontaneous short-lived sub-state openings (Zhang et al., 2021). This result suggested that it may require the removal of fewer lipids to activate MscS in the presence of DPPC. Indeed, while a βCD concentration of 750 μM failed to elicit any MscS activity in a pure azolectin membrane, it resulted in robust activation of MscS if the membrane contained 15% DDPC (Fig. 4C). For MscL, the Gly22Ser mutation reduces the activation threshold of the channel to close to that of MscS (Yoshimura et al., 1999). In this case, a vCD concentration of 5 mM failed to elicit any activity for wild-type MscL, but activated multiple Gly22Ser mutant channels (Fig. 4D).

While CDs have so far only been used for functional assays using patch-clamp electrophysiology (a system that already allows for easy manipulation of membrane tension through pressure application to the membrane patch), it is likely that they will also be useful for functional assays in systems for which it is less straightforward to apply a defined mechanical perturbation, such as droplet interface bilayers, supported droplet bilayers and planar lipid bilayers as well as for liposome flux assays. Equally exciting is the prospect of using CDs to activate FFL-based MS channels in living cells, although biological effects would have to be carefully confirmed to be indeed mediated by the activation of MS channels rather than other effects caused by depleting the plasma membrane of lipids (for example by repeating the experiment in the presence of the MS channel blocker GsMTx4, which should then no longer result in biological effects).

In conclusion, CDs have recently been introduced as a new tool to induce tension in membranes that makes it possible to study the effect of membrane tension on MS channels both structurally and functionally (Fig. 5). While initial results already suggest that CDs could potentially be used to activate all FFL-gated MS channels, CDs could have many more applications, for example to study other MS membrane proteins, such as integrins and certain G protein-coupled receptors. Since CDs have different specificities for membrane lipids, it may also be possible to use CDs to study membrane proteins that are regulated by specific lipid species, in particular cholesterol, which can be preferentially extracted by methylated βCD.

**Fig. 5:**
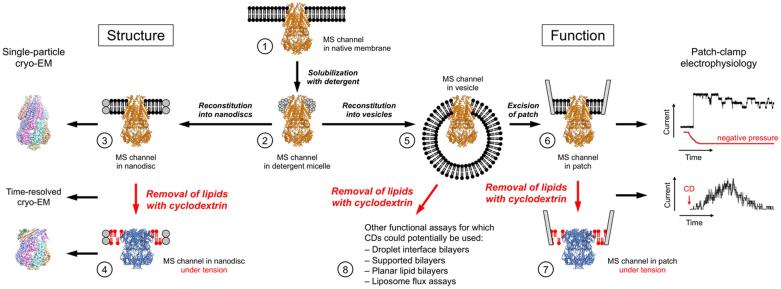
The use of CDs in structural and functional studies of MS channels. (1) The proper function of MS channels that are gated based on the FFL principle depends critically on the presence of a native lipid bilayer environment. (2) Solubilization of MS channels with detergent makes them amenable to structure determination by X-ray crystallography, but it can remove functionally important lipids and the detergent micelle is an imperfect substitution for a native lipid bilayer. (3) Reconstitution of MS channels with lipids into MSP-based nanodiscs allows for the use of single-particle cryo-EM to determine the structure of the channel in the context of a lipid bilayer. (4) Incubation of the nanodiscs with CDs will remove lipids and the effect of the resulting membrane tension on the embedded MS channel can be visualized by cryo-EM. Note that time-resolved structural studies are possible by freezing samples after different incubation periods and/or by using different CD concentrations. (5) For functional studies, the detergent-solubilized MS channel can be reconstituted into liposomes that allow for vesicle-based functional studies. (6) Most commonly, patches are excised from the liposome to perform electrophysiological studies, in which MS channels have typically been activated by the application of negative pressure. (7) Alternatively, the membrane patch can be perfused with CDs, which will remove lipids, generating membrane tension that will activate the embedded MS channels. (8) CDs may also be useful to create membrane tension in a variety of other functional assays, including in other electrophysiological recording systems, in which it is currently difficult to control membrane tension (e.g., droplet interface bilayers, planar bilayers), and in fluorescence-based approaches such as liposome flux assays.

## CRediT authorship contribution statement

**Yixiao Zhang:** Writing – review & editing. **Gabriella Angiulli:** Visualization, Writing – review & editing. **Boris Martinac:** Writing – review & editing. **Charles D. Cox:** Supervision, Writing – review & editing. **Thomas Walz:** Supervision, Visualization, Writing – review & editing.

## Declaration of Competing Interest

The authors declare that they have no known competing financial interests or personal relationships that could have appeared to influence the work reported in this paper.
